# Changing pattern of infectious agents in postneurosurgical meningitis

**Published:** 2014

**Authors:** Davood Yadegarynia, Latif Gachkar, Alireza Fatemi, Alireza Zali, Niloufar Nobari, Mitra Asoodeh, Zahra Parsaieyan

**Affiliations:** 1 Infectious Diseases and Tropical Medicine Research Center, Shahid Beheshti University of Medical Sciences, Tehran, Iran

**Keywords:** * Acinetobacter Baumannii*, Post Neurosurgical Meningitis, Pattern, Infectious Agents

## Abstract

***Background: ***The rates of postneurosurgical infections, particularly meningitis and the most common pathogens have been reported variable during the recent years. The aim of this research was to determine the prevalence of postneurosurgical meningitis and its current spectrum of infectious agent.

***Methods:*** In a descriptive study, the postneurosurgical patients’ cerebrospinal fluid was cultured on blood and MacConkey agar plates and evaluated at Microbiology Laboratory. 55 significant isolates as species level were recognized by bacteriological techniques.

***Results:*** *Acinetobacter baumannii (A. baumanni*i) was the most common organism [29(52.7%)], followed by *Klebsiella pneumoniae* [8(14.5%)], *Methicillin-resistant Staphylococcus aureus* [6(10.9%)], *Staphylococcus saprophyticus* [5(9.1%)], *Pseudomonas aeruginosa* [3(5.5%)], *Methicillin-sensitive Staphylococcus aureus* [3(5.5%)], and *Enterococcus faecalis* [1(1.8%)]. The majority of *A. baumannii* isolates were carbapenem-resistant.

***Conclusion: ***Our research revealed that the rate of postneurosurgical meningitis due to carbapenem-resistant *A. baumannii* has been increased. This finding emphasized the importance of preventive strategies against *A. baumannii*. The changing pattern of infectious agents in postneurosurgical meningitis over time suggests the necessity of other studies.

Neurosurgical procedures, for example, craniotomy, cerebrospinal fluid (CSF) shunting, and external ventricular drain (EVD) insertion may lead to central nervous system (CNS) infections. Meningitis is the most common infectious complication caused by the different kinds of pathogens (1, 2). This complication is rare with accompanying high morbidity and mortality without immediate treatment (2). The rates of postneurosurgical meningitis have been reported from 0.3% to 10% (1-7). Because of subtle and unusual manifestations, nonspecific CSF parameters, and aseptic meningitis due to meningeal inflammation after neurosurgical procedures, the definite diagnosis is challenging (8, 9). Furthermore, some studies have shown the high rates of negative CSF smears or cultures in patients with bacterial meningitis (10, 11). Previous studies found some risk factors for postneurosurgical CNS infections, such as implantation of foreign body, CSF leakage, previous neurosurgical infection, absence of antibiotic prophylaxis, duration of surgery over 4 hours, interventions involving nasal sinuses, emergency surgeries, and prior to radiation therapy (2, 3, 12). 

The most common pathogen in many reports was *Staphylococci*, particularly *Staphylococcus aureus *(1-3, 7, 9, 13-15), whereas some recent reports have shown the higher rates of *gram-negative* organisms (10, 11, 16). The changing pattern of pathogens necessitates more research. Also, the locally published data are lacking. Thus, we conducted this study to determine the prevalence of postneurosurgical meningitis and its current spectrum of infectious agents in a private hospital in Tehran, Iran.

The aim of this research was to determine the prevalence of postneurosurgical meningitis and its current spectrum of infectious agents.

## Methods

We conducted a descriptive study from March 2010 through March 2012 in a private hospital in Tehran, Iran. We selected the patients that underwent neurosurgical procedures, followed by fever, headache, loss of consciousness, or meningismus. Then, the patients’ CSF was obtained and inoculated into blood and MacConkey agar plates at the Microbiology Laboratory. We incubated these plates at 37°C for 24 hours, and recognized the significant isolates as species level by common bacteriological techniques. The negative cultures were excluded. Totally, 55 pathogens were isolated at the Microbiology Laboratory from the patients’ specimens. The control organisms used were *Staphylococcus aureus* ATCC 29213 and 25923*[non-Methicillin-resistant Staphylococcus aureus (non-MRSA)]*; ATCC 43300 *(MRSA)*; *Enterococcus faecalis* ATCC 51299; *Staphylococcus epidermidis* ATCC 12228; *Staphylococcus saprophyticus* ATCC BAA-750; *Enterococcus faecalis *ATCC 29212; *Streptococcus pneumoniae* ATCC 49619; *Escherichia coli* ATCC 35218; *Klebsiella pneumoniae* ATCC 700603; *Pseudomonas aeruginosa* ATCC 27853; *Acinetobacter baumannii* ATCC BAA-747; and *Enterobacter aerogenes* ATCC 13048 to control the quality of media and evaluate color stability. Three control American Type Culture Collection (ATCC) organisms were used for each test, and we examined each test for each new group varied (17). Susceptibility pattern was determined by disk diffusion method based on Kirby-Bauer method on Mueller-Hinton agar plate for gram-negative pathogens (18). The following disks were used: Amoxicillin clavulanate (30 μg/disk) was placed in the center of plate while ceftriaxone (30 μg/disk), cefixime (5 μg/disk), cefotaxime (30 μg/disk), carbenicillin (100μg/disk), cephalexin (30μg/disk), trimethoprim-sulfamethoxazole (25μg/disk), amikacin (30 μg/disk), gentamicin (10 μg/disk), ciprofloxacin (5 μg/disk), imipenem (10 μg/disk), tetracycline (30 μg/disk), nalidixic acid (30 μg/disk), piperacillin-tazobactam (110 μg/disk), and colistin (10 μg/disk) were situated around the central disc. Five disks were been placed on each plate. The Clinical and Laboratory Standards Institute (CLSI) recommends a zone size of ≥ 5mm difference between the cephalosporin disk with and without clavulanic acid which is considered as significant and Extended Spectrum Beta Lactamase (ESBL) producing bacteria (19). For *Staphylococcus aureus* isolates, methicillin and vancomycin susceptibility were determined by using the CLSI microdilution methods (BHI). Portion (10μg) of 0.5 McFarland suspensions were pipetted onto brain heart infusion agar plates (17). 

The following data obtained in patients with positive CSF cultures: Age, gender, ward, type of neurosurgery, the use of device in CNS, the days of meningeal signs and symptoms appearance after the neurosurgery, type of organism, antimicrobial susceptibility, and outcome. 

## Results

From March 2010 through March 2012, 1593 patients underwent neurosurgical procedures in our hospital. 90% of patients had a kind of device (68% EVD, 20% Shunt, and 1% Prosthesis). 176(11%) patients were suspected to have postneurosurgical meningitis (fever, headache, loss of consciousness, or meningismus). 55 patients (3.5%) had positive CSF culture. 31 patients were males (56%) and 24 were females (44%). The mean age of patients was 32.4±20.9. The youngest patient was 1 year of age, the oldest 78. Most of the patients belonged to the young age group between 20-29 years (20%) (table 1).

28 patients (51%) had an emergency neurosurgery. 44 patients (80%) were admitted to Intensive Care Unit (ICU), and the others were hospitalized in neurosurgery ward. The mean days of the average time from onset of meningeal signs and symptoms after surgery was 1.3±0.5 (Minimum 1 day and maximum, 3 days). All patients received antibiotics before the signs and symptoms appear. As shown in table 2, craniotomy in terms of the neurosurgical procedures, accounted for the greatest number [47(85.5%)]. 16.5% of Prosthesis, 13.5% of Spinal fusions, 13% of CSF Shunts, 11% of Laminectomies, 9.5% of Craniotomies, 7.5% of EVDs, and 3% of Discectomies led to meningitis. In meningitis group, a kind of device was used for 48(87.3%) patients. Most of them were EVD [31(56.4%)], followed by CSF shunt [16(29.1%)], and prosthesis [1(1.8%)].

**Table 1:** The Frequency (Percentage) of Patients’ Age Group in Post Neurosurgical Meningitis

**Age Group**	**Frequency**	**%**
<10 years	10	18.2
10-19 years	7	12.7
20-29 years	11	20
30-39 years	10	18.2
40-49 years	5	9.1
50-59 years	4	7.3
≥60 years	8	14.5
Total	55	100

**Table 2:** The Frequency (Percentage) of Neurosurgical Procedures in Patients with Post Neurosurgical Meningitis

**Procedure**	**Frequency**	**Percentage**
Craniotomy	47	85.5
Spinal Fusion	3	5.5
Laminectomy	3	5.5
Discectomy	2	3.5
Total	55	100

*Acinetobacter baumannii* was the most common organism [29(52.7%)], followed by *Klebsiella pneumoniae* [8(14.5%)], *MRSA* [6(10.9%)], *Staphylococcus saprophyticus* [5(9.1%)], *Pseudomonas aeruginosa* [3(5.5%)], *Methicillin-sensitive Staphylococcus aureus (MSSA)* [3(5.5%)], and *Enterococcus faecalis* [1(1.8%)].

67% of g*ram-negative bacilli* isolates were ESBL. 76% of *A. baumannii* isolates were carbapenem-resistant (only resistant to colistin) and the others (24%) were multi-drug resistant (only sensitive to carbapenems and colistin). 62% of *A. baumannii* infections led to death. 2 patients with *A. baumannii* infection had recurrence. 

Unfortunately, the mortality rate was 45%. 57% of infections due to g*ram-negative bacteria* were companied with death, whereas, only two patients (13%) with g*ram-positive* infection died. 28 patients (51%) were treated, and 2 (3.5%) of them had a recurrence. 

## Discussion

In our study, the incidence of postneurosurgical meningitis was 3.5%, which has been similar to the recent reports (11, 16). All patients received prophylactic antibiotics after the surgery. Several articles have noted the importance of this matter (2, 11). The rate of emergency surgeries in McClelland and Hall (31%) and Logigan et al.’s studies (16%) was remarkably lower than our research (51%) (2, 15). McClelland and Hall showed 1.1% of craniotomies, 1.6% of CSF shunts and none of laminectomy, discectomy, and spinal fusion led to meningitis (2). In Logigan et al.’s study, 55% and 32% patients with postneurosurgical meningitis underwent craniotomy and CSF shunt insertion, respectively (15). We found the intermediate rates: 9.5% of craniotomies, 13% of CSF shunts, 13.5% of spinal fusions, 11% of laminectomies, and 3% of discectomies.

In this study, g*ram-negative bacilli*, particularly *A. baumannii* was the most common organism. The most common pathogen in Van de Beek et al. (1), McClelland and Hall (2), Korinek et al. (3,13), Erman et al. (7), Zarrouk et al. (9), Apisarnthanarak et al. (14), Logigan et al.’s (15) reports was *Staphylococci*, particularly *Staphylococcus aureus*, whereas some recent reports have shown the higher rates of *gram-negative* organisms (10, 11, 16, 20).

The present study showed that 76% of *A. baumannii* isolates were carbapenem-resistant. These rates were remarkably higher than Gaynes and Edwards (21), Rahbar and Hajian (22), Metan et al. (23), Logigan et al. (15), Baek-kim et al. (24), Cascio et al. (25), Vahdani et al. (26), Aminzadeh and Yaghubi (27), Khan et al. (28), and Moon et al.’s (29) findings. Mortality rate varies in different studies from 5% to 39% (8, 15, 16, 28-30). Unfortunately, the mortality rate of our patients was higher (45%). The majority of meningitis caused by *A. baumannii* led to death. 

Our research and other studies (21-29) revealed that the rate of postneurosurgical meningitis due to *A. baumannii* and its resistance to broad-spectrum antibiotics, particularly to carbapenems has been increased. Carbapenem-resistant *A. Baumannii* is a matter of great concern in terms of treatment (23-25, 27). The major predisposing factors are previous carbapenems administrations, ICU admittance, emergency neurosurgeries, total parenteral nutrition, using device, and long duration of hospitalization (31, 32). Baek-Kim showed that the postneurosurgical infections due to *A. baumannii* are more resistant (24). Inappropriate use of antibiotics as a prophylaxis or empirical treatment, contamination of operation room, ICU, or laboratory, and changing pattern of infectious agents in postneurosurgical meningitis are the possible reasons that necessitate further research (10).

In conclusion, in our study we found a high rate of post neurosurgical meningitis due to *Acinetobacter baumannii *resistant to carbapenems. This finding emphasizes the importance of preventive strategies against *gram-negative bacilli*, especially *A. baumannii*. The changing pattern of infectious agents in postneurosurgical meningitis over time suggests the necessity of other studies to give the most up-to-date insight to physicians.

## Notes

**Funding: **This work was supported by Infectious Diseases and Tropical Medicine Research Center, Shahid Beheshti University of Medical Sciences, Tehran, Iran.
